# Editorial: Nutritional and Environmental Modulation of the Endocrine System: Effects on Metabolism and Growth

**DOI:** 10.3389/fendo.2019.00354

**Published:** 2019-06-04

**Authors:** Oliana Carnevali, Encarnación Capilla

**Affiliations:** ^1^Department of Life and Environmental Sciences, Polytechnic University of Marche, Ancona, Italy; ^2^Department of Cell Biology, Physiology and Immunology, University of Barcelona, Barcelona, Spain

**Keywords:** growth, metabolism, endocrine disruptors, feed factors, appetite regulation

Metabolism and growth are under the control of the endocrine system that, working in cooperation with the nervous system, regulates these functions. The objective of this Research Topic was to provide a multidisciplinary approach of cutting-edge research on metabolism and growth in different experimental models, including farmed species. The Research Topic contains 12 contributions, comprising 7 original research articles, 3 reviews, and 2 minireviews. These works include a wide range of cellular and *in vivo* models, methodological and conceptual approaches. The Topic focused on recent research conducted in the field of metabolism and growth, and aimed to address key questions about the interplay between nutritional, environmental, or other external factors (i.e., temperature or pollutants) and the endocrine system, as well as the modulation of signals involved in the control of feed intake, regulating these processes.

From fish to mammals, the growth hormone (GH)/insulin-like growth factor I (IGF-I) axis is the major endocrine system stimulating growth (1, 2), indicating a strong evolutionary conservation. GH regulates growth directly, but also indirectly through induced production of IGF-I, mostly in the liver, but also in peripheral tissues, where this growth factor exerts paracrine and autocrine actions. In the current Topic, Pérez-Sánchez et al. reviewed the evolution of the GH, prolactin, and somatolactin family of peptides and their sub-functionalization in marine fish species, and revisited the direct and indirect effects of GH and IGF-I on growth and development. In addition to growth, the GH/IGF-I system controls other physiological functions including metabolism and mineral balance, and, to properly grow and meet metabolic demands, hormones produced from the brain and peripheral tissues contribute regulating feeding (3, 4). The GH/IGF-I system and the hormones controlling appetite are regulated by both, internal and external signals. The internal signals inform about the metabolic status of the organism and in relation to that, Pérez-Sánchez et al. commented on sirtuins, new markers informing of energy status that can modulate the anabolic actions of GH. In this line, Björnsson et al. evaluated the impact of energy reserves on GH resistance and circulating GH-binding protein levels in rainbow trout (*Oncorhynchus mykiss*) of different genetic background, demonstrating that initial body reserves affect whether or not GH resistance is acquired under catabolic physiological conditions. In another study in pregnant women treated with *Lycium barbarum* L. polysaccharides (LBLP), a relation between insulin resistance and secretion, lipid profiles, and miR-33 levels was reported, showing the beneficial effects of LBLP improving the symptoms of gestational diabetes mellitus (Yang et al.). Moreover, the close relationship between growth and reproduction has been discussed in a manuscript focusing on the low levels of leptin associated with the overexpression of GH in transgenic common carp (*Cyprinus carpio*) and the resulting delay in puberty onset (Chen et al.).

Among the external signals modulating growth and metabolism, diet availability and composition are the most notable. In line with this, one review within the Topic has explored the nutritional factors that through influencing the endocrine system control feeding and growth in fish, describing the nutrients as well as the brain and peripheral hormones that regulate food intake (Bertucci et al.). Secondly, the recent knowledge achieved concerning the interaction of stress with the different neurosensing mechanisms and peripheral endocrine signals such as leptin that regulate food intake in fish have been revised (Conde-Sieira et al.). Then, considering the effects of specific nutrients on metabolism, the physiological and epigenetic changes induced by methionine restriction have been reviewed encompassing both, mammalian and fish models (Neslund Latimer et al.). Next, specifically in gilthead seabream (*Sparus aurata*), the effects of dietary supplementation with the aminoacid derivative creatine on muscle growth and quality, have been explored (Ramos-Pinto et al.). Increasing dietary creatine enlarged dorsal cross-sectional muscle area and modulated the expression of genes related to muscle growth (i.e., myogenic regulatory factors, MRFs) and texture (i.e., protein degradation), although further studies are required to better clarify these effects in fish.

Considering environmental signals, two articles within the Topic have explored the effects of temperature on metabolism and growth in different fish species. Sanhueza et al. reported in Atlantic salmon (*Salmo salar*), that a wide thermal range is associated with significant increases in gene transcription of circadian-clock related genes and monoamines hormone levels, which decrease aggressive behavior, and positively influence stress, welfare, and growth, whereas a restricted thermal range showed the opposite effects. Moreover, since climate change is a major challenge that humanity is facing nowadays, a study investigated in gilthead sea bream, the effects of three rearing temperatures (19, 24, and 28°C) on growth and lipid metabolism using *in vivo* and *in vitro* approaches (Balbuena-Pecino et al.). Increasing temperatures caused unfavorable musculoskeletal growth conditions due to reduced expression of GH/IGF-I system members and specific MRFs genes. Moreover, to cope with the increased energy needs, lipid metabolism was induced in the muscle although not efficiently.

Besides abiotic factors, environmental contaminants are known to cause adverse health effects, impairing not only reproduction but also metabolism and development in wildlife and humans, since there is evidence that endocrine disruptive chemicals (EDCs) can interact with a variety of hormones and/or hormone receptors, exerting actions as agonists or antagonists (5, 6). In the present Topic, the effects of EDCs on lipid metabolism and health and their potential “transgenerational obesogenic effects” have been revised, focusing on the modifications occurring at hepatic level in different animal models (Maradonna and Carnevali). Moreover, the effects of several EDCs individually and in mixture were tested on male fathead minnows (*Pimephales promelas*) through analysis of liver transcriptomes by expression microarrays and subsequent quantitative PCR, identifying distinct modes of action for the different chemicals as well as pinpointing a number of new biomarkers that can potentially be used for environmental screening (Zare et al.).

Taken together, these studies have demonstrated the effects of dietary and environmental factors on metabolic processes, and the complex interactions of all these factors on the control of the endocrine system regulating growth and metabolism mainly in model organisms and farmed species.

## Author Contributions

OC and EC drafted and critically reviewed the article.

### Conflict of Interest Statement

The authors declare that the research was conducted in the absence of any commercial or financial relationships that could be construed as a potential conflict of interest.
